# Roles of TGFβ and FGF signals during growth and differentiation of mouse lens epithelial cell *in vitro*

**DOI:** 10.1038/s41598-017-07619-5

**Published:** 2017-08-04

**Authors:** Dong Wang, Eddie Wang, Kelsey Liu, Chun-hong Xia, Song Li, Xiaohua Gong

**Affiliations:** 1 School of Optometry and Vision Science Program, University of California Berkeley, California, 94720 USA; 2 Department of Bioengineering, University of California, Berkeley, California, 94720 USA; 3 Department of Bioengineering, University of California, Los Angeles, California, 90095 USA; 4 Department of Medicine, University of California, Los Angeles, California, 90095 USA

## Abstract

Transforming growth factor β (TGFβ) and fibroblast growth factor (FGF) signaling pathways play important roles in the proliferation and differentiation of lens epithelial cells (LECs) during development. Low dosage bFGF promotes cell proliferation while high dosage induces differentiation. TGFβ signaling regulates LEC proliferation and differentiation as well, but also promotes epithelial-mesenchymal transitions that lead to cataracts. Thus far, it has been difficult to recapitulate the features of germinative LECs *in vitro*. Here, we have established a LEC culture protocol that uses SB431542 (SB) compound to inhibit TGFβ/Smad activation, and found that SB treatment promoted mouse LEC proliferation, maintained LECs’ morphology and distinct markers including N-cadherin, c-Maf, Prox1, and αA-, αB-, and β-crystallins. In contrast, low-dosage bFGF was unable to sustain those markers and, combined with SB, altered LECs’ morphology and β-crystallin expression. We further found that Matrigel substrate coatings greatly increased cell proliferation and uniquely affected β-crystallin expression. Cultured LECs retained the ability to differentiate into γ-crystallin-positive lentoids by high-dosage bFGF treatment. Thus, a suppression of TGFβ/Smad signaling *in vitro* is critical to maintaining characteristic features of mouse LECs, especially expression of the key transcription factors c-Maf and Prox1.

## Introduction

Cataracts are opacities of the lens and are the leading cause of world blindness according to the World Health Organization^1^. Although a cataractous lens can be surgically removed and replaced with an artificial lens, many patients cannot get this treatment, especially in developing countries. Therefore, non-surgical treatments or preventative therapies for cataracts are needed, but sorely lacking. Therapeutic development may require improved lens cell culture models in order to better understand the mechanisms of lens transparency and cataractogenesis.

The vertebrate lens is surrounded by a basement membrane capsule, beneath which lies a monolayer of lens epithelial cells (LECs) on the anterior hemisphere and a bulk mass of elongated fiber cells. Throughout life, LECs in a region just anterior to the lens equator known as the germinative zone are the most prone to proliferation and subsequent differentiation into lens fibers^2^. After intraocular lens implantation, undesired LEC proliferation, differentiation and migration can cause secondary cataracts. Therefore, a thorough understanding of LEC biology is required to understand lens growth, differentiation, and disease.

LECs were first cultured from embryonic chicken by Kirby in 1927^3^. Since then, chicken LECs cultures have been widely used and significantly improved upon, especially by Menko *et al*.^4, 5^ and Musil *et al*.^6^. For mammalian LEC culture, Mann was the first to culture mouse embryonic LECs and observe cell growth^7^. After that, human fetal LECs were cultured, which expressed the epithelial marker αB-crystallin and the differentiating fiber cell markers β- and γ-crystallins^8, 9^. However, LECs isolated from different species including rat^10^, calf^11^, monkey^12^, and human^13–16^, grew slowly *in vitro*, and spontaneously differentiated into fiber cells or underwent apoptosis^17^. Several LEC lines established from rabbit or murine lenses lack appropriate expression of crystallins and their abilities to form differentiated lentoid bodies are uncertain^18–20^. Immortalized LEC lines generated with recombinant viruses^21–23^ were unable to differentiate into lentoids or lens fibers. Explant culture is an excellent alternative for mammalian lens culture in which LECs are grown on their native basement membrane capsule in serum free media conditions^24, 25^. However, explants display restricted cell proliferation, the majority of LECs on explants remain to have cell-cell contact inhibition, such that the most proliferative cells are presumably present at the explant’s edges. For research that benefits from high throughput techniques and large cell numbers, such as drug screening, lens capsule explant culture may not be an optimal choice, due to the large number of donor lenses that would be required and limited number of cells per lens (e.g. 40,000–50,000 LECs on the mouse lens capsule^26, 27^). At present, it is difficult to expand and maintain distinct properties of mammalian LECs *in vitro* due to epithelial-mesenchymal transitions (EMT) and cell senescence.

Results from previous studies have demonstrated the importance of basic fibroblast growth factor (bFGF) and transforming growth factor β (TGFβ) on LEC behavior^2, 28–30^. The capsule explant model was used to show that low concentrations bFGF could maintain LEC proliferation, while high concentrations could promote fiber cell differentiation and lentoid formation^31, 32^. Further studies show that the TGFβ signaling pathway plays an important role in EMT and the secondary cataract development^33, 34^. Under stimulation with TGFβ1, LECs lost epithelial identity, underwent EMT, differentiated into myofibroblast-like cells, or underwent apoptosis^33–36^. Phosphatidylinositol 3-OH kinase (PI3K)/Akt signaling and Snail are necessary for TGF-β induced EMT in LECs^37^. Inhibition of the TGFβ pathway with the TGFβ type 1 receptor inhibitor, SB431542, prevented or reversed EMT thereby maintaining epithelial identity in various cell types, including human keratinocytes and murine renal tubular epithelial cells^38–40^.

In this study, we examined the effects of SB431542, bFGF, and Matrigel coating on dissociated mouse LECs in a new medium. We found that a culture condition with SB431542 treatment promoted the proliferation of mouse LECs and supported some identities of lens equatorial epithelial cells *in vitro*. Low dosage bFGF treatment and Matrigel coating further promoted LEC proliferation, but affected cell morphology and/or expression of β-crystallins. The cultured LECs from mature lenses could be differentiated into lentoids by high dosage bFGF treatment. This culture condition provides an excellent alternative system for a uniform expansion of mouse LECs *in vitro*, which provides sufficient cells for investigating LEC proliferation, fiber cell differentiation and the regulation of crystallins expression (especially, β-crystallin isoforms) without undesired EMT or apoptosis.

## Results

### Inhibition of TGFβ signaling with SB431542 is critical for mouse primary cultured LECs *in vitro*

**Figure 1:**
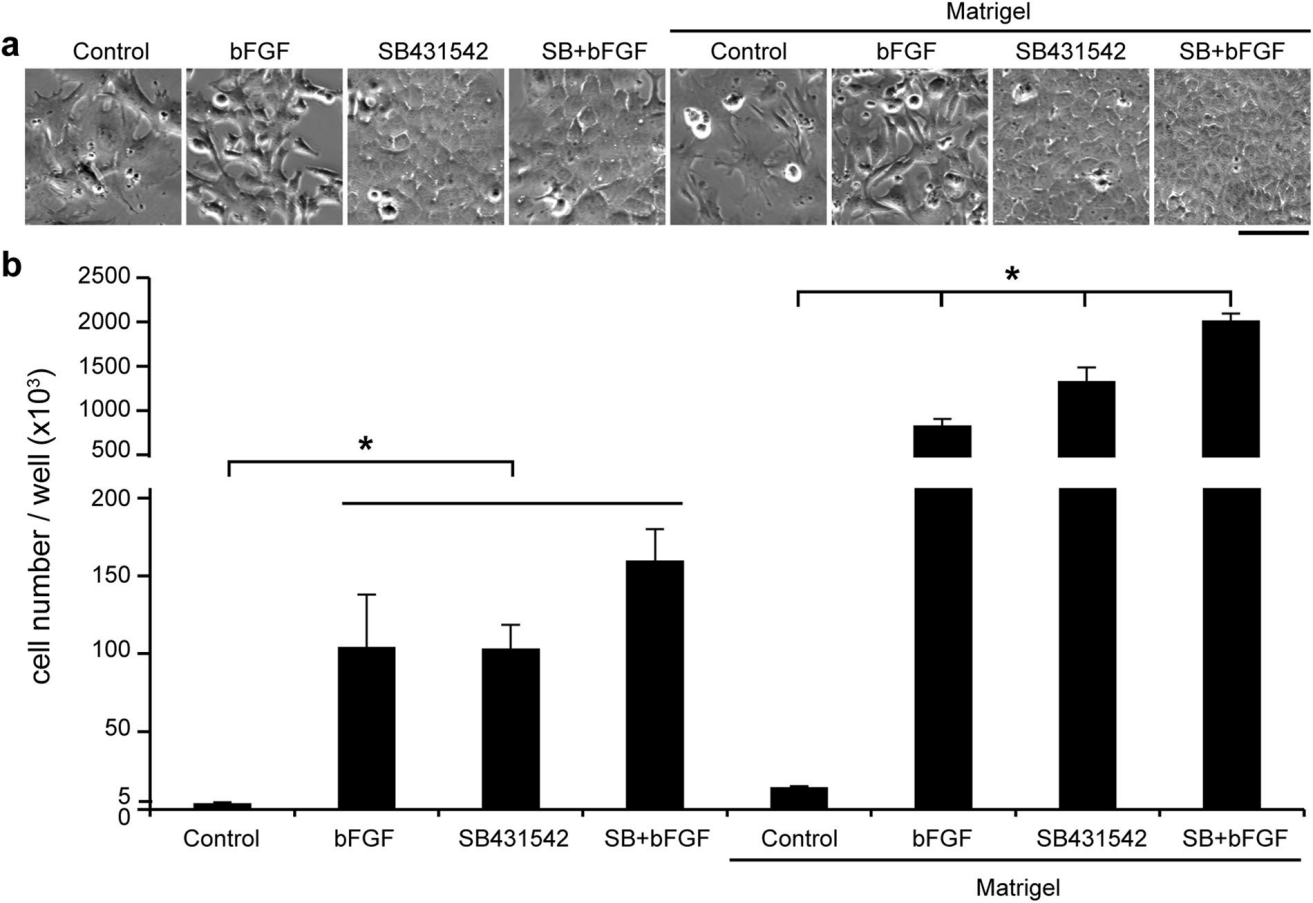
Treatment with SB431542 alone maintains adult mouse LEC morphology and promotes cell growth *in vitro*. (**a**) Phase contrast images of adult mouse LECs that were cultured on dishes with or without Matrigel coating, in media supplemented with nothing (Control), 5 ng/ml bFGF, 5 µM SB431542, or SB431542 and bFGF (SB + bFGF). (**b**) Primary adult mouse LECs were cultured in 6-well plates at a seeding density of 5000 cells per well. The cell counts for each group were determined after one week. N = 3. Scale bar, 100 µm. Two-way ANOVA showed a significant interaction between media composition and Matrigel coating. Post-hoc testing showed that all Matrigel coated conditions were significantly greater than their corresponding uncoated conditions. For uncoated conditions, bFGF, SB, and SB + bFGF groups were not significantly different than each other but were all significantly greater than control. For the coated conditions, all media resulted in significantly different cell counts than each other coated condition. *P < 0.05.

Mouse LECs were isolated from 3–4 week old lenses and cultured under different combinations of bFGF (5 ng/ml) and SB431542 (5 µM), and with or without Matrigel coated substrates (Fig. 1). We found that the LECs in the control groups grew very slowly and had a myofibroblast-like morphology (Fig. 1a), suggesting EMT. Only LECs treated with SB431542 maintained the cuboidal-like cell morphology. Surprisingly, SB431542 treatment alone was also sufficient to promote LEC growth to a similar extent as bFGF treatment did (Fig. 1b). Matrigel coating greatly promoted cell growth, but SB431542 treatment was still required for maintaining the cuboidal-like cell morphology. These data suggest that inhibition of TGFβ signaling with SB431542 is important to maintain cuboidal cell shape and proliferation of LECs *in vitro*.

**Figure 2:**
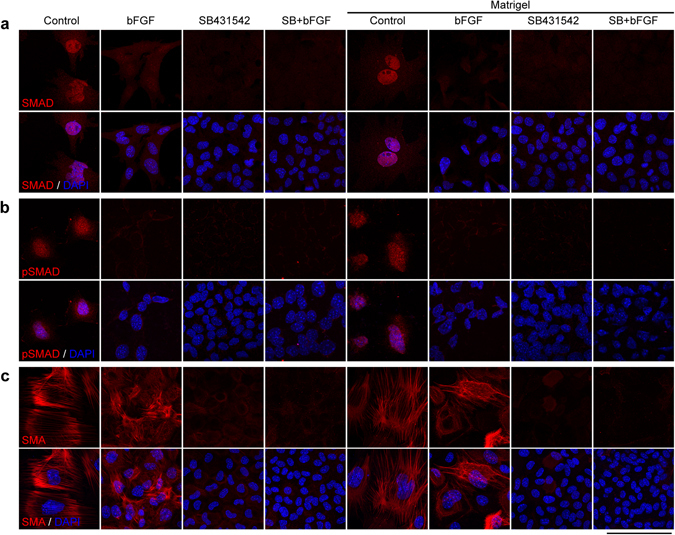
Inhibition of TGFβ signaling by SB431542. Mouse LECs of different culture groups were immunostained by the antibodies against SMAD2/3 (**a**), pSMAD2/3 (**b**) and SMA (**c**). Cell nuclei were stained by DAPI. Scale bar, 100 µm.

To examine whether there was active TGFβ signaling in the control groups that underwent EMT and whether TGFβ signaling was inhibited by SB431542, we performed immunofluorescence imaging. The results showed SMAD2/3 and phosphorylated SMAD2/3 signals in the nuclei and strong smooth muscle α-actin (SMA) in the cytoplasm of control LECs, but no or very weak signals in the LECs treated with SB431542 (Fig. 2). It was interesting that bFGF treatment also inhibited TGFβ signaling as shown by very weak SMAD2/3 and phosphorylated SMAD2/3 in the nuclei, but had SMA expression, which suggests that bFGF alone was not sufficient to inhibit EMT and maintain the cuboidal morphology of LECs (Figs 1 and 2).

### LECs cultured with SB431542 recapitulate lens equatorial epithelial cells marker expression

**Figure 3:**
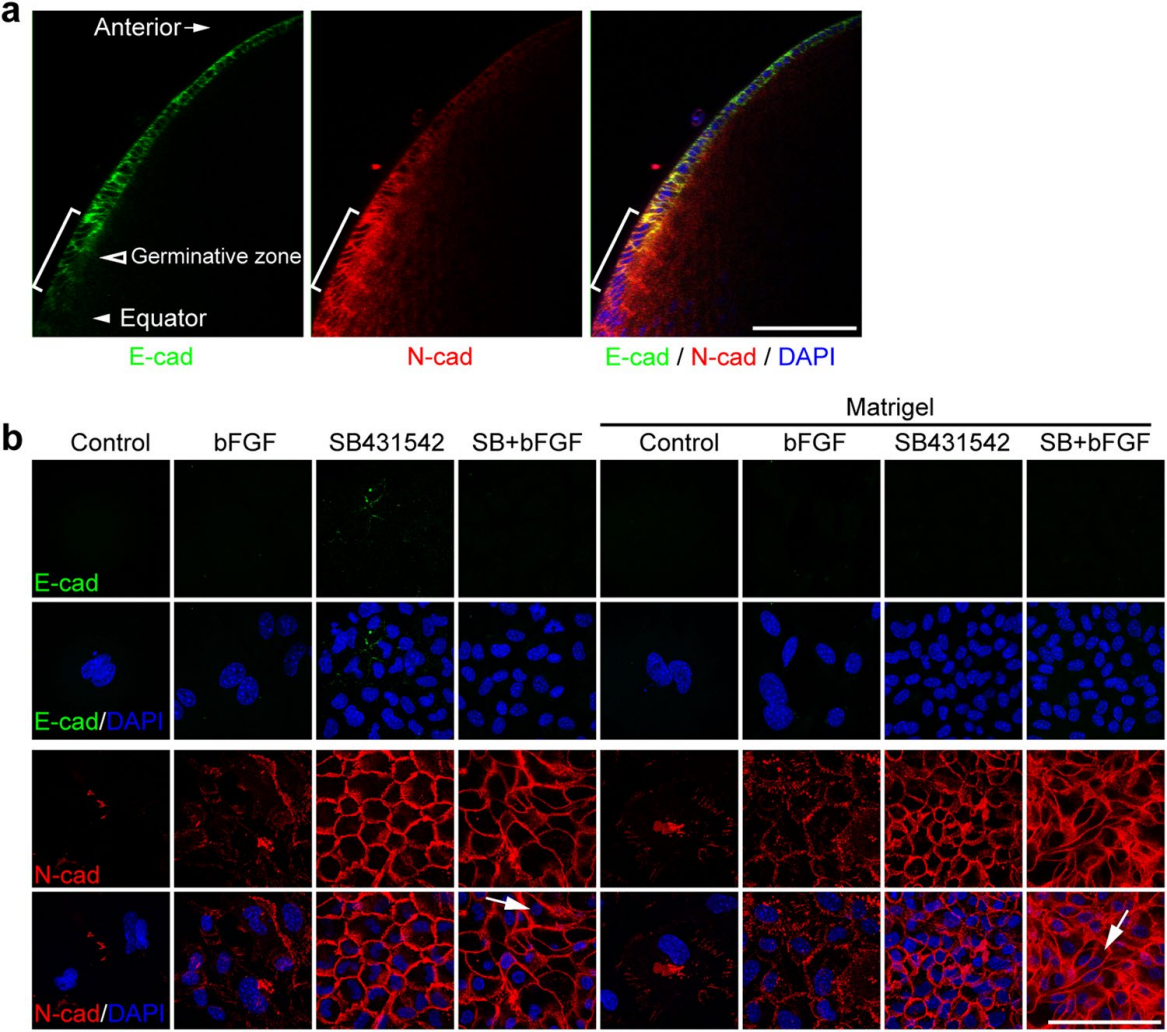
Treatment with SB431542 maintains N-cadherin expression in mouse LECs. (**a**) Whole-mounted mouse lens stained by antibodies against E-cadherin and N-cadherin. (**b**) Adult mouse LECs were cultured in different conditions for one week, and immunostained by antibodies against E-cadherin and N-cadherin. Cell nuclei were stained by DAPI. Brackets in a: transitional zone. Arrows in (**b**) elongated cells. Scale bars, 100 µm.

In a stained, intact mouse lens, we observed that E-cadherin expression decreased and N-cadherin expression increased when following LECs from the anterior to the germinative zone and lens equator (Fig. 3a). Immunostaining results of LECs under different culture conditions revealed that LECs lacked obvious E-cadherin expression (Fig. 3b). Few LECs under the control media condition expressed N-cadherin. The majority of bFGF treated LECs had N-cadherin expression to some extent, but it was not uniformly distributed at cell boundaries. All SB431542 treated cuboidal-like LECs displayed uniform distribution of N-cadherin on their cell boundaries. LECs treated with both bFGF and SB431542 showed high expression of N-cadherin, but many cells displayed deformed or elongated cell shape (Fig. 3b). Matrigel coated conditions had no dramatic effects on E-cadherin and N-cadherin expression and cell morphology compared to uncoated conditions (Fig. 3b). LECs without SB431542 treatment often displayed myofibroblast-like cell shapes with enlarged nuclei, expression of strong SMA and reduced expression of N-cadherin, indicating a loss of distinct epithelial cells features (Figs 1–3). These results indicate that these cultured mouse LECs share some distinct features with germinative zone LECs. However, there are obvious differences between these cultured LECs *in vitro* and germinative zone LECs *in vivo*, shown below.

### Presence of SB431542 is essential to maintain the expression of c-Maf and Prox1 but not Pax6

**Figure 4:**
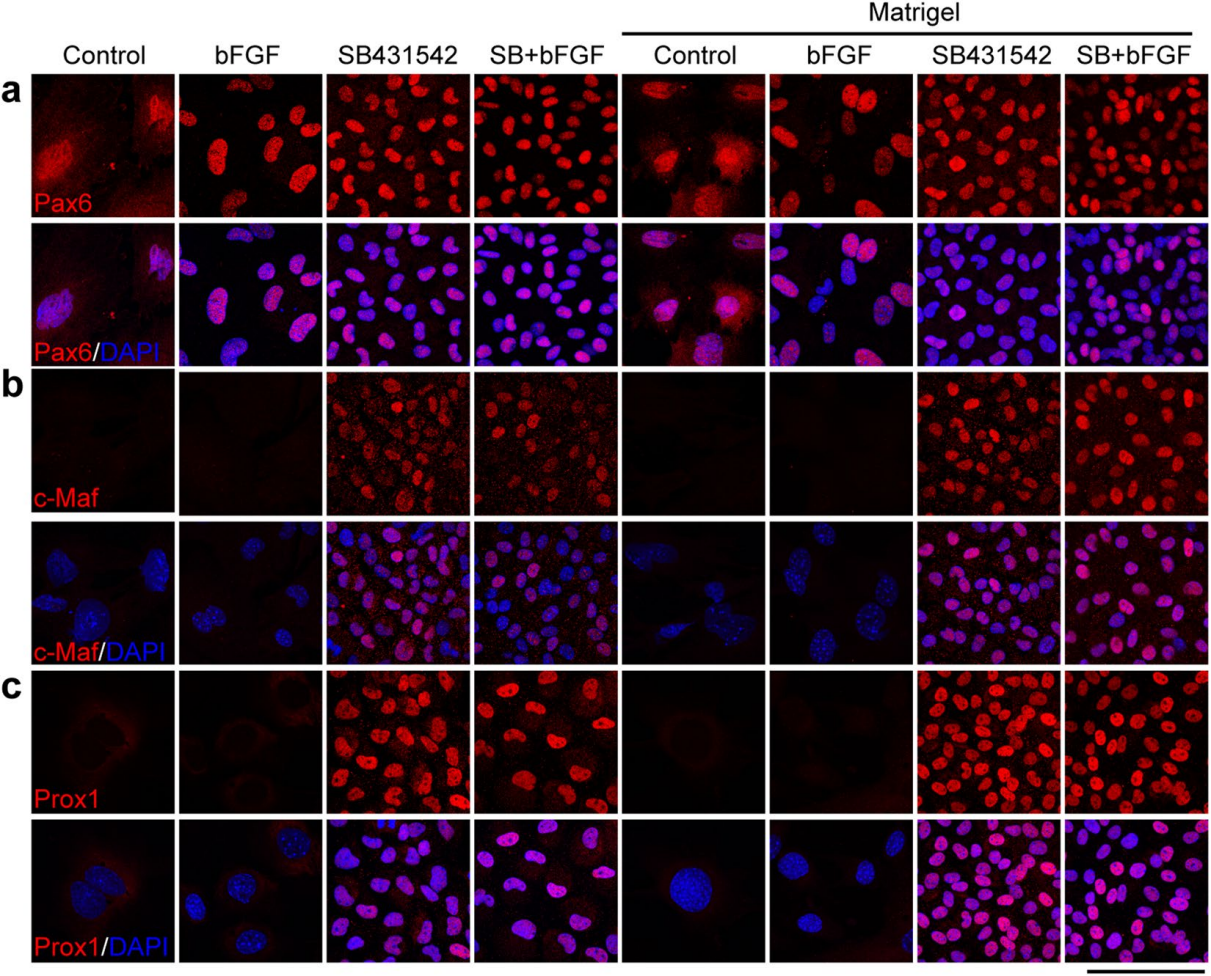
Treatment with SB431542 maintains c-Maf and Prox1 expression in adult mouse LECs. Mouse LECs of different groups were cultured for one week and immunostained by antibodies against Pax6 (**a**), c-Maf (**b**), and Prox1 (**c**). Cell nuclei were stained by DAPI. Scale bar, 100 µm.

We next examined lens-specific transcription factors to further characterize LEC identity in different culture conditions. Specific transcription factors such as Pax6, c-Maf, and Prox1 are important in determining the cell fate, proliferation and differentiation of LECs^41^. Pax6 was detected under all culture conditions (Fig. 4a), but both c-Maf and Prox1 were uniformly detected in cells with SB431542 treatment regardless with or without FGF (Fig. 4b,c). The control and bFGF treatment alone were insufficient to support the expression of c-Maf and Prox1. Matrigel coating had no obvious effect on the expression profiles of these transcription factors (The right part of the panels in Fig. 4). Thus, SB431542 treatment is sufficient to support the expression of c-Maf and Prox1 in cultured mouse LECs.

### SB431542 treatment promotes the expression of αA-, αB- and β-crystallin in mouse LECs

**Figure 5:**
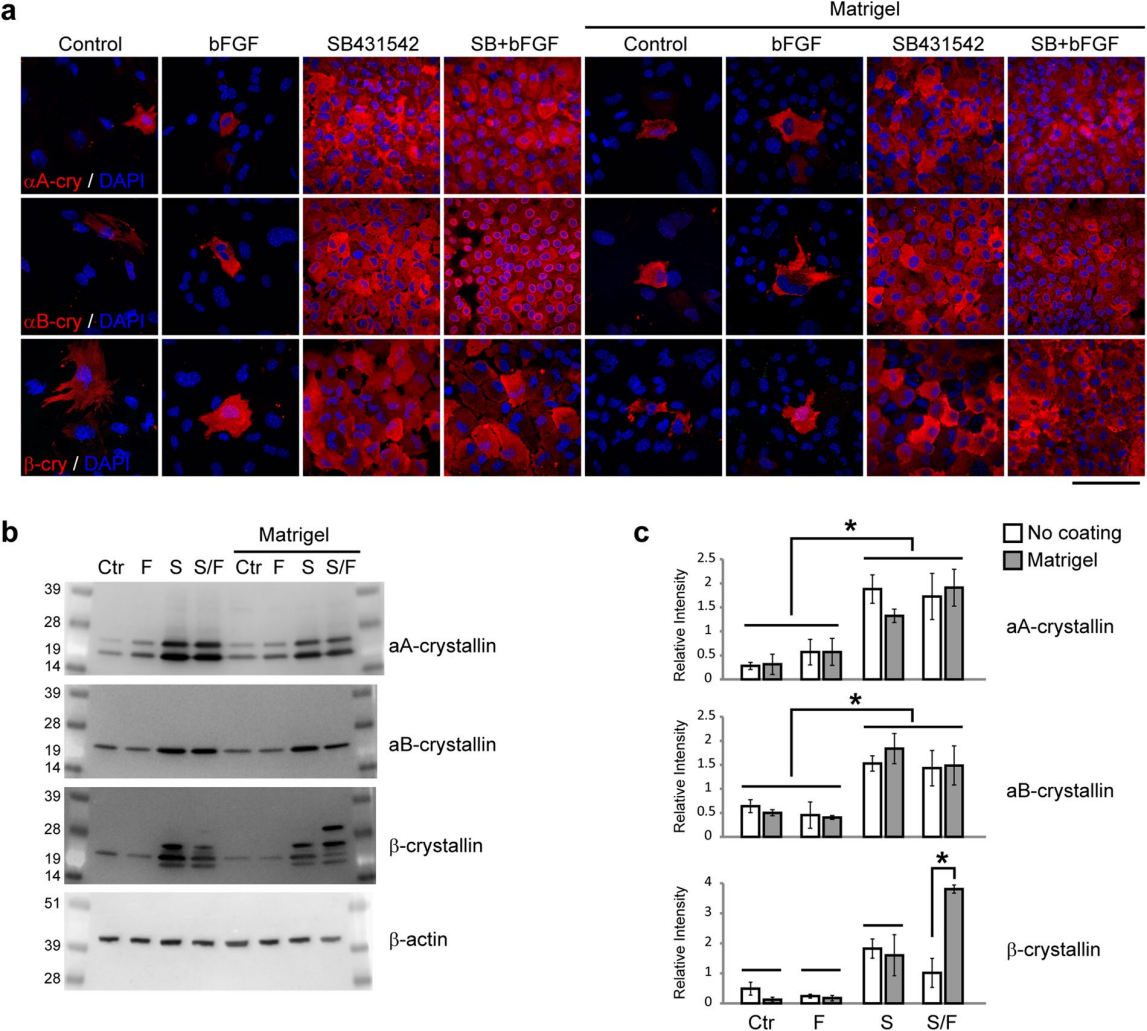
Treatment with SB431542 maintains αA-, αB- and β-crystallin expression in mouse LECs. (**a**) Mouse LECs were cultured in different conditions for one week, and immunostained by antibodies against αA-, αB- and β-crystallin. Cell nuclei were stained by DAPI. (**b**) Western blot results showed that proteins of primary adult mouse LECs in each culture condition were probed by the antibodies against αA-, αB-, β-crystallin, and β-actin. (**c**) Two-way ANOVA showed that only media composition, was important to αA- and αB-crystallins expression. Post-hoc tests showed that Control and bFGF media had less protein expression than SB and SB+bFGF media. β-crystallin levels showed an interaction between media composition and coating. Unlike αA- and αB-crystallins, Matrigel coating combined with SB and FGF significantly increased β-crystallin levels compared to SB+bFGF without coating and other coated conditions. Scale bar, 100 µm. N = 3. *P < 0.05.

The expression profiles of different crystallin proteins such as αA-, αB- and β-crystallin are useful markers for monitoring distinct properties of LECs in proliferation and differentiation. We examined both the distribution and level of different crystallins in LECs by immunostaining and western blotting. Very few LECs in control and bFGF treated samples regardless with or without Matrigel coating had αA-, αB- and β-crystallins (Fig. 5a). On the contrary, almost all the cells with SB431542 treatment, regardless with or without bFGF, or Matrigel coating, showed the presence of αA-, αB- and β-crystallins (Fig. 5a). Western blot data confirmed these immunostaining observations and showed that SB431542 treatment elevated the expression of different β-crystallin isoforms in both uncoated and Matrigel coated conditions (Fig. 5b,c). Without Matrigel coating, LECs with SB431542/bFGF treatment had reduced β-crystallin expression when compared to those with SB431542 treatment alone (p value, 0.036). With Matrigel coating, βB1-crystallin (the largest band detected in western blotting^42, 43^) was detected only in the LECs with SB431542/bFGF treatment (Fig. 5b). These results indicate that SB431542 treatment is critical to maintaining the expression of αA-, αB- and β-crystallin in cultured LECs and Matrigel coating may affect the expression of βB1-crystallin under certain conditions.

### Differentiation of LECs into lentoids

**Figure 6:**
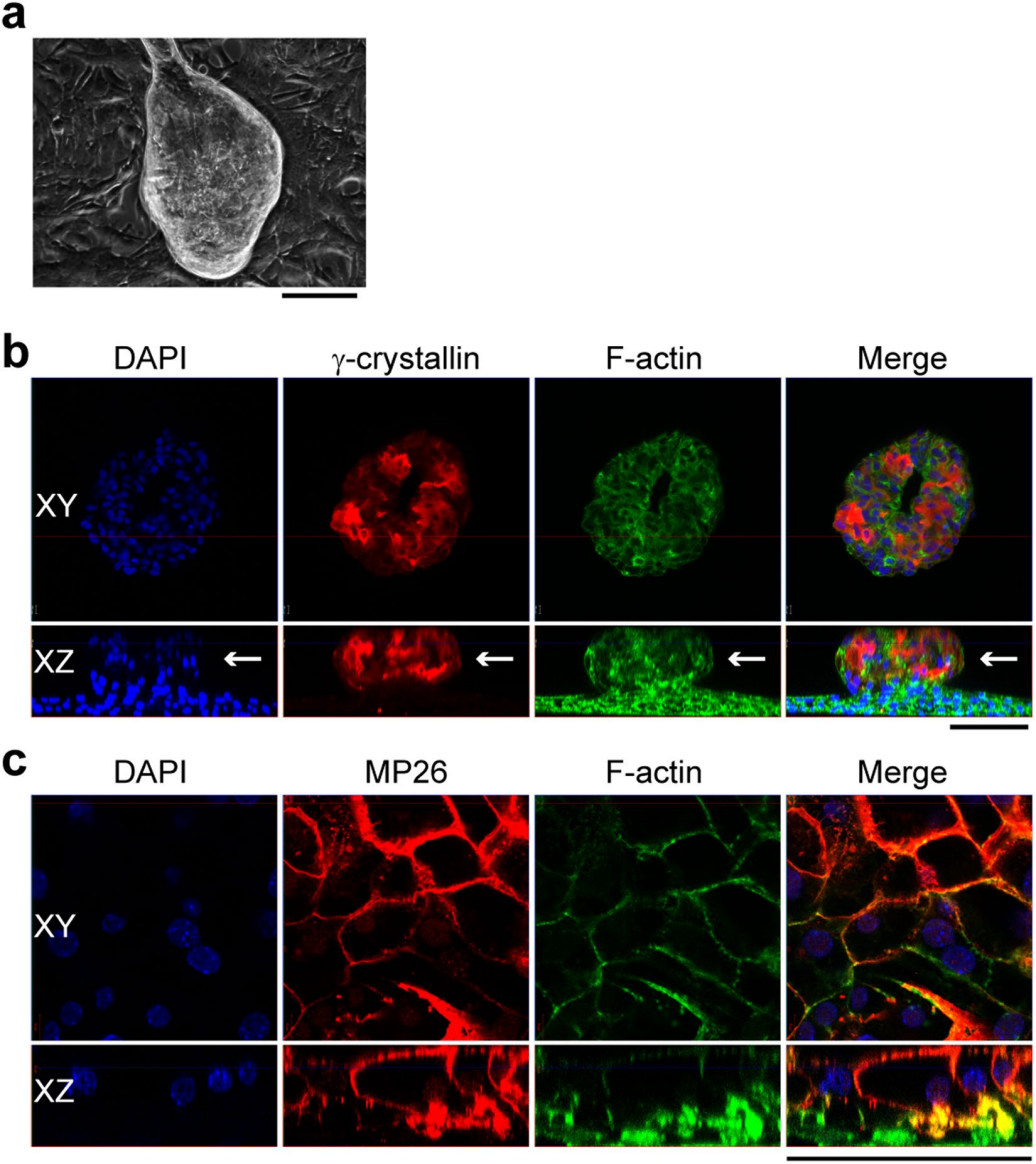
Mouse LECs could be induced to form lentoids *in vitro*. (**a**) Phase contrast image of lentoid after three weeks of treatment with 200 ng/ml bFGF. The lentoids were immunostained with antibodies against γ-crystallin (**b**) and MP26 (**c**). Cell nuclei were stained by DAPI and F-actin was stained by phalloidin. White arrow, the lentoid from side view. Scale bar, 100 µm.

We next examined whether LECs could still differentiate into lentoid bodies. The LECs treated with SB431542 and Matrigel coating were used for differentiation assays. The primary cultured LECs were passaged to Matrigel-coated dishes at a density of 10^5^ cells per 35-mm dish. The medium supplemented with SB431542 was used in the first two days of culture, and then changed to medium supplemented with 200 ng/ml high bFGF, without SB431542. Large lentoid bodies were observed after two to three weeks of high bFGF induction (Fig. 6a) and had varied sizes with diameters of 100–400 µm (See Supplementary Fig. S1). The total surface area of lentoid bodies was about 19 ± 5% of culture dishes. These lentoids expressed fiber cell markers including γ-crystallin and MP26 (Fig. 6b,c). Thus, these LECs have retained the capacity to differentiate into lens fiber-like cells.

## Discussion

We found that SB431542 treatment, an inhibition of TGFβ/Smad signaling, is essential for maintaining distinct properties of primary cultured mouse LECs *in vitro*. These cultured mouse LECs express many specific markers of lens equatorial epithelial cells, including the adhesion molecule N-cadherin^44^, lens-specific transcription factors c-Maf and Prox1, and αA-, αB- and β-crystallins^41^. Moreover, the proliferation of LECs can be further promoted by Matrigel coating, which partly mimics certain components of lens capsule. The culture conditions with SB431542 treatment and Matrigel coating is highly repeatable and allows one mouse lens containing about 40,000–50,000 cells^26^, to be expanded into about 6 million cells after 7–10 days in culture. These LECs can be differentiated into lentoid forming lens fibers under high dosage bFGF. Thus, we have overcome difficulties such as EMT and senescence associated with mouse LEC culture *in vitro*. This LEC culture system provides a complementary approach to lens explant culture systems for studying the proliferation and differentiation of mammalian LECs.

This work suggests that inhibition of TGFβ/Smad pathway is critical for maintaining the proliferation and distinct identity of mouse LECs *in vitro*. TGFβ is known to have both negative and positive effects on cell proliferation depending on the tissue or cell type^45, 46^. It induced epithelial growth arrest and apoptosis in mammary, skin, colon, pancreas, liver and prostate, but had opposite effects on cell proliferation in different cell layers of skin^46^ and affected acinar cell differentiation during pancreas development^47^. TGFβ signaling is essential for normal lens development and lens fiber differentiation^48^. TGFβs exist in ocular media and their activities are regulated by inhibitors, such as α2-macroglobulin^49^ and HtrA3^50^. If not regulated properly, TGFβ could contribute to EMT and cataract formation^36^. In lens capsule explant culture models, addition of TGFβ1 to the culture media induced LECs to adopt a mesenchymal cell shape, secrete excessive extracellular matrix, differentiate into myofibroblasts, undergo apoptosis and lose lens cell identity^33, 34^. Inhibiting TGFβ by α2-macroglobulin maintained LEC morphology *in vitro* ^49^. SB431542 is a small molecule inhibitor of TGFβ type I receptor^40^, and could restore cell identity in other epithelial cells *in vitro* ^37–39^, but had never been fully characterized in lens epithelial cells. This work reveals that suppression of TGFβ/Smad pathway with SB431542 promotes mouse LEC proliferation, and supports cell morphology and the expression of c-Maf and Prox1.

Previous reports showed that a low concentration of bFGF promotes LECs proliferation and high bFGF promotes differentiation^2, 51^. bFGF can also counteract TGFβ-induced EMT and apoptosis in rat lens capsule explant culture^52^. Here we confirm that a low concentration of bFGF (5 ng/ml) promotes the proliferation of mouse LECs, suppressed TGFβ/Smad signaling, but at the same time is insufficient to inhibit SMA expression and maintain several key mouse LECs markers such as αA/αB-crystallins, c-Maf, and Prox1. Also, with bFGF alone, some cells might undergo EMT and adopt elongated cell shapes. Moreover, some cells might differentiate to highly express αA-, αB-, β-crystallins. Matrigel coating does not affect the consequence of low bFGF culture conditions. However, combined treatments of bFGF with SB431542 or bFGF with SB431542 and Matrigel coating alter the expression of β-crystallin isoforms in different manners. This culture system may be useful for studying the regulation of β-crystallin isoforms by FGF and other signaling pathways.

Matrigel is an extracellular protein mixture extracted from Engelbreth-Holm-Swarm mouse sarcoma, containing mainly collagen IV and laminin, and has been used for *in vitro* culture of various cell types. The lens capsule is composed of several extracellular proteins including collagen IV, laminin and fibronectin, and functions as a basement membrane for LEC attachment, proliferation, migration and differentiation^53^. Lens cells have membrane bound integrins that bind extracellular matrix proteins and regulate cell proliferation and differentiation^54^. Here, we observed that LECs in the Matrigel coating group had about 10 times the cell numbers compared to those without Matrigel coating. The effects on primary cultured mouse LECs match the roles of extracellular matrix *in vivo*, but differ from a previous study that showed that extracellular matrix proteins promoted human LEC line attachment and survival, but not proliferation^55^. This discrepancy indicates that this mouse LEC culture system may be a better model to study the mechanisms of lens epithelial cell proliferation *in vitro* than the virus-transfected human lens cell lines altered with unknown changes.

Pax6 is a master transcription factor during mammalian lens development^41^, and its expression in LECs is directly negatively regulated by TGFβ/SMAD signaling pathway^56, 57^. Pax6 expression is similar in all culture conditions including the control without bFGF and SB431542, which suggests maintained autoregulation of Pax6^56^. Pax6 controls expression of transcription factors including c-Maf and Prox1^58^ and α-crystallin^59^. Surprisingly, both c-Maf and Prox1 are barely detectable without SB431542. C-Maf is highly expressed in the equatorial lens cells and activates α- and β-crystallin expression^60^, and Prox1 regulates lens fiber differentiation^61^. Presence of both c-Maf and Prox1 induced by SB431542 treatment promotes the expression αA-, αB- and β-crystallins as a part of mechanism to maintain the characteristic features of lens equatorial epithelial cells *in vitro*.

In conclusion, inhibition of TGFβ pathway by the small molecule SB431542 promotes LEC proliferation and maintains characteristic features of mouse LECs *in vitro*. Matrigel coating further improves LEC proliferation. These LECs can be induced to differentiate into lentoids by high concentrations of bFGF. This *in vitro* model provides sufficient cells from an individual animal to study mouse LEC proliferation and differentiation. This model also provides a useful platform for studying lens epithelial cells in pathological conditions and for seeking potential therapeutic targets for cataract prevention. For future work, there are several challenges to overcome: (1) This culture method utilizes FBS and Matrigel that contain small amounts of growth factors with variations between batches. More defined culture protocols will be needed in the future for knowing all growth factors in the medium. The downstream signaling activities of insulin-like growth factor (IGF), platelet-derived growth factor (PDGF) and other growth factors are unknown in cultured mouse LECs. Perhaps, testing IGF and PDGF dosages in serum free medium will be a future direction for developing a defined culture medium. (2) Although this method can successfully expand primary mouse LECs, they undergo senescence after two passages. Further modifications are also needed for translational studies in human LECs. (3) Instead of malformed lentoids, defined 3D lens culture models mimic native microenvironments *in vitro* by using mouse or human LECs will be valuable for investigating fiber differentiation and testing therapeutic reagents.

## Materials and Methods

### Mouse Strains

Mouse care, breeding, and euthanasia were performed according to an Animal Care and Use Committee (ACUC)–approved animal protocol (University of California, Berkeley, CA, USA) and the ARVO Statement for the Use of Animals in Ophthalmic and Vision Research. Lenses of wild-type 129S4 (129-SvJae) mice at the ages of 3–4 weeks were used for LEC culture.

### Primary Lens Cell Culture

Lenses were immediately dissected from enucleated eyeballs of euthanized mice. After removing surrounding tissue, lenses were incubated in 0.05% trypsin-EDTA (Gibco, cat#: 25300-054) at 37 °C for 10 min to further remove any non-lens cells and then transferred to a new dissection tray. Lens capsules were peeled off by forceps and transferred into 100 µl of dispase per pair of lenses (2 U/ml, Sigma, cat#: D4693) in Advanced DMEM-F12 medium (Gibco, cat#: 12634-010). After 5 min of dispase treatment, 100 µl of 10 × TrypLE (Gibco, cat#: A12177-02) was added. After 10 min, the cell suspension was transferred to a new sterile tube and spun down at 1000 rpm for 4 min. The cell pellet was resuspended in culture medium and seeded into culture dishes. The control culture medium (Ctr) was Advanced DMEM/F12, supplemented with 1 × Penicillin-Streptomycin (Gibco, cat#: 15140-122), 1 × GlutaMax (Gibco, cat#: 35050-061), 2% Fetal Bovine Serum (FBS, Gibco, cat#: 26140-079), and 1 × B-27 (Gibco, cat#: 17504-044). To test the effects of bFGF (Peprotech, cat#: 100-18B) and SB431542 (Stemgent, cat#: 04-0010-10), the control medium was further supplemented with 5 ng/ml bFGF (F), 5 µM SB431542 (S), or 5 ng/ml bFGF plus 5 µM SB431542 (SF). SB431542 was dissolved in DMSO to make stock solutions. Equal volumes of DMSO without SB431542 were added as vehicle controls to the Ctr and FGF groups. For Matrigel coating, the dishes were coated with Matrigel (Mg, 1:100 in Advanced DMEM/F12, Corning, cat#: 354248) for at least one hour at 37 °C, and washed with Advanced DMEM/F12 medium before seeding cells. A total of 8 different culture conditions (Ctr, F, S, SF, Mg-Ctr, Mg-F, Mg-S, and Mg-SF) were tested at 37 °C with 5% CO_2_ and 95% humidity incubation.

### Proliferation and Differentiation of Primary Cultured LECs

To assay cell proliferation, 5000 cells per well were seeded into 6-well plates with 3 wells per experimental group. Culture medium was changed every day. After one week, total cell number per well was counted.

As differentiation medium, Advanced DMEM/F12 supplemented with 1× Penicillin-Streptomycin, 1× GlutaMax, 2% FBS, 1× B-27, and 200 ng/ml bFGF, was used and changed daily.

### Western Blot

After one week of culture, LECs were collected in lysis buffer containing 20 mM Tris (pH 7.5), 150 mM NaCl, 1 mM EDTA, 1 mM EGTA, 1% Triton X-100, and protease inhibitor cocktail (Roche, Cat#: 11 836 153 001). The cell suspension was sonicated for 10  seconds by a Kontes micro ultrasonic cell disrupter, and then centrifuged at 14,000 rpm for 10 min. The supernatant was used for experiments. Protein concentration was measured by Coomassie assay (Pierce, cat#: 23200). Equal volumes of 2× sample buffer (0.1 M Tris-PO_4_, pH 6.8, 4% SDS, 8% Bromophenol blue, 20% Glycerol, 10% 2-mercaptoethanol) was added to the protein solution and mixed well. All procedures were performed on ice. Equal amounts of protein (10 µg) were separated by SDS-PAGE and transferred to PVDF membranes. The membranes were blocked with 5% non-fat milk and incubated with antibodies against αA-, αB-, β-crystallin, or β-actin (Sigma, cat#: A5441), at 4 °C overnight. After three 5 min washes in TBST (0.15 M NaCl, 0.05 M Tris, pH 7.4, 0.1% Tween 20) buffer, the membranes were incubated in HRP-conjugated secondary antibodies for one hour. After three washes with TBST, protein bands were visualized by an Azure Biosystems c600 imager.

### Statistical analysis

Data were reported as means ± standard deviation (s.d.) All experiments were repeated at least three times. To account for the increases in variance with increases in cell counts, cell counts were transformed by putting them to the 1/4th power. Two-way ANOVA was performed on the transformed data followed by Tukey’s Honest Significant Difference (HSD) comparisons. For western blot analysis, two-way ANOVA was performed followed by Tukey’s HSD comparisons. p-values < 0.05 were considered significant.

### Immunostaining

Cultured mouse LECs after one week of culture and lentoids after three weeks of treatment with 200 ng/ml bFGF were fixed in 4% paraformaldehyde (PFA) in Phosphate Buffered Saline (PBS) for 15 min and then washed with PBS 3x, blocked with 5% normal goat serum in 0.3% Triton-X100 for one hour, and were incubated with specific primary antibodies diluted in blocking buffer overnight at 4 °C. For pSMAD2/3 staining, cells were permeabilized with methanol prior to blocking. The primary antibodies included SMA (Sigma, cat#A2547), SMAD2/3 (BD, cat#610843), pSMAD2/3 (Cell Signaling, cat#8828 S), αA-, αB- and γ-crystallin (generously provided by Dr. Joseph Horwitz at Jules Stein Eye Institute), β-crystallin (generously provided by J. Samuel Zigler at the National Eye Institute), Pax6 (Santa Cruz Tech, cat#sc-53108), c-Maf (Bethyl Laboratories, cat#: A300-613A), and Prox1 (EMD Millipore, cat#: AB5475), MP26 (generously provided by Joseph Horwitz at the Jules Stein Eye Institute of UCLA). After incubation with primary antibodies, the cells were washed with PBS and incubated in secondary antibodies (Jackson ImmunoResearch Laboratories) diluted in blocking buffer for one hour. The cells were then placed in mounting medium containing DAPI (Vector Laboratories, cat#: H-1200) and imaged by confocal microscopy (Zeiss, LSM700).

For whole-mount staining, mouse lenses were fixed in 1% PFA at 4 °C overnight and then washed with PBS 3 × 30 min on a shaker, blocked with 5% normal goat serum in 0.3% Triton X-100 for 2 h, incubated in primary antibodies (E-cadherin, Invitrogen cat#13-1900; N-cadherin, Invitrogen, cat#33-3900) diluted in blocking buffer at 4 °C for 1 day. The lenses were then washed with PBS three times for 30 min on a shaker, and then incubated in secondary antibodies diluted in blocking buffer at 4 °C overnight, washed with PBS 3 × 30 min, incubated in DAPI solution diluted in PBS for 1 h, and then imaged by confocal microscopy (Zeiss, LSM700).

## Electronic supplementary material

Supplementary information
