# Supplementary material for: Roles of TGFβ and FGF signals during growth and differentiation of mouse lens epithelial cell in vitro
Source: Sci Rep. 2017 Aug 4;7:7274. doi: 10.1038/s41598-017-07619-5 (PMC5544739; doi:10.1038/s41598-017-07619-5)
Supplement: Supplementary file 1 — Supplementary information [file 41598_2017_7619_MOESM1_ESM.pdf]

Roles of TGFβ and FGF signals during growth and differentiation of mouse lens epithelial cell *in vitro*

Dong Wang[1,2,3], Eddie Wang[1], Kelsey Liu[1], Chun-hong Xia[1], Song Li[2,3,4] & Xiaohua Gong[1,*]

[1] School of Optometry and Vision Science Program, University of California Berkeley, California 94720, USA

[2] Department of Bioengineering, University of California, Berkeley, California 94720, USA

[3] Department of Bioengineering, University of California, Los Angeles, California 90095, USA

[4] Department of Medicine, University of California, Los Angeles, California 90095, USA

* Corresponding author, Xiaohua Gong, Ph.D., School of Optometry and Vision Science Program, University of California, Berkeley, 695 Minor Hall, Berkeley, CA 94720. Email: xgong@berkeley.edu

Supplementary Information: Figure S1.

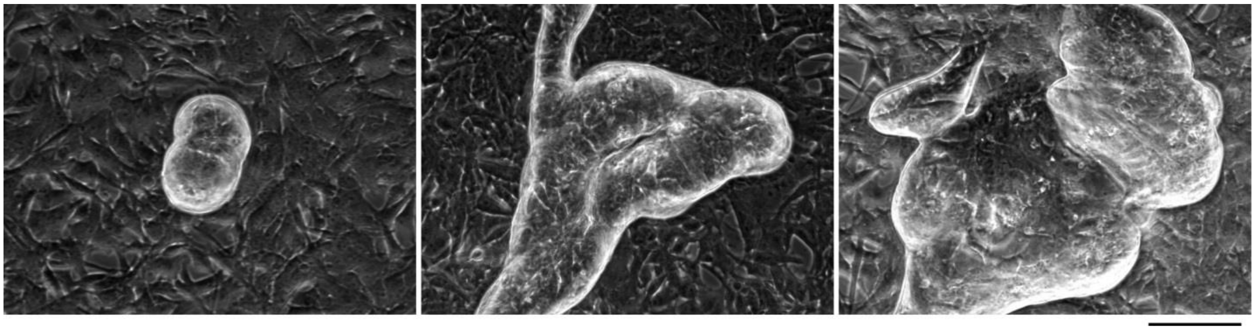

Supplementary Figure S1. Phase contrast images of lentoid bodies of different sizes. Scale bar, 100 µm.
